# Characterization of Detailed Audiological Features of Cytomegalovirus Infection: A Composite Cohort Study from Groups with Distinct Demographics

**DOI:** 10.1155/2018/7087586

**Published:** 2018-08-30

**Authors:** Bong Jik Kim, Jae Joon Han, Seung Han Shin, Han-Suk Kim, Hye Ran Yang, Eun Hwa Choi, Mun Young Chang, Sang-Yeon Lee, Myung-Whan Suh, Ja-Won Koo, Jun Ho Lee, Byung Yoon Choi, Seung-Ha Oh

**Affiliations:** ^1^Department of Otolaryngology-Head and Neck Surgery, Chungnam National University, College of Medicine, Daejeon 35015, Republic of Korea; ^2^Department of Otolaryngology, Seoul National University Bundang Hospital, Seoul National University, College of Medicine, Seongnam 13620, Republic of Korea; ^3^Department of Pediatrics, Seoul National University Hospital, Seoul National University, College of Medicine, Seoul 03080, Republic of Korea; ^4^Department of Pediatrics, Seoul National University Bundang Hospital, Seoul National University, College of Medicine, Seongnam 13620, Republic of Korea; ^5^Department of Otolaryngology, Chung-Ang University, College of Medicine, Seoul 06973, Republic of Korea; ^6^Department of Otolaryngology, Seoul National University Hospital, Seoul National University, College of Medicine, Seoul 03080, Republic of Korea

## Abstract

Congenital cytomegalovirus (cCMV) infection is a common congenital infection that causes sensorineural hearing loss (SNHL). Despite its substantial impact on public health and cost burden, epidemiology and clinical features of CMV-related SNHL have never been reported in the Korean populations. This study investigated the detailed audiologic phenotypes of cCMV infection to see if a specific SNHL pattern is associated with a particular clinical setting. A total of 38 patients with cCMV infection were studied retrospectively. Patients were classified into three groups with distinct demographics: clinically driven diagnosis (n=17), routine newborn CMV screening according to the NICU protocols (n=10), or referral to ENT for cochlear implant (CI) (n=11). The incidence of cCMV infection was 3.6%, showing 33.3% of SNHL among cCMV patients, 38% of asymmetric hearing loss, 29% of late-onset hearing loss, and diverse severity spectrum in patients with CMV-related SNHL. CI recipients with CMV-related SNHL showed a significantly improved speech perception. Surprisingly, in 36.4 % of CI implantees, initial audiological manifestation was significant asymmetry of hearing thresholds between both ears, with better ear retaining significant residual hearing up to 50dB. CMV turns out to be a significant etiology of SNHL, first to date reported in the Korean pediatric population. Analysis of audiologic phenotypes showed a very wide spectrum of SNHL and favorable CI outcomes in case of profound deafness. Especially for the patients with asymmetric hearing loss, close surveillance of hearing should be warranted and CI could be considered on the worse side first, based on the observation of rapid progression to profound deafness of better side.

## 1. Introduction

Congenital cytomegalovirus (cCMV) infection is a common congenital infection with an incidence rate ranging from 0.5 to 2% of all live births [1, 2]. Children with cCMV infection are born with or develop permanent disabilities, including sensorineural hearing loss (SNHL), vision loss, and neurodevelopmental delay, with SNHL being the most common manifestation [3].

Since CMV was first identified as a cause of deafness in 1964 [4], it has been reported that CMV infection accounts for approximately 40% of nongenetically caused congenital SNHL, representing about 20% of all congenital SNHL. This signifies that CMV infection is the leading cause of congenital hearing loss with significant public health costs. There are two types of cCMV infection, symptomatic and asymptomatic, based on the presence of clinical manifestation at birth. Approximately 10% of neonates with cCMV infection are symptomatic; they are born with clinically apparent sequelae, including SNHL, mental retardation, motor disability, and microcephaly (symptomatic cCMV infection) [5]. The remaining 90% are asymptomatic at birth (asymptomatic cCMV infection). However, about 6-23% of neonates with asymptomatic cCMV infection can also develop late-onset SNHL, while 33-63% of symptomatic patients can have SNHL [3, 6, 7].

Previously, maternal seroprevalence of CMV has been shown to be able to predict the prevalence of cCMV in infants; every 10% increase in maternal seroprevalence corresponds to 0.26% in CMV at birth [1]. Given the relatively high CMV maternal seroprevalence (98.1%) in Korea [8], the incidence of cCMV infection in the Korean population is likely not low. Although its associated public health and cost burden have well been documented, there is, to the best of our knowledge, a lack of studies evaluating cCMV infection in the Korean population. To date, only one, single-institution study reported the incidence of cCMV infection in Korea, which was shown to be 1.2% [9]. Furthermore, to our knowledge, there has not been any study investigating CMV-related hearing loss in the Korean population.

To date, diagnosing CMV-related SNHL has been a challenge to many clinicians due to the short time window for making the correct diagnosis and relatively low awareness of disease entity by pediatricians and otolaryngologists. However, CMV-related SNHL is one of the few etiologies of preventable and treatable hearing loss in children. Here, we investigated the epidemiology of cCMV infection-related SNHL and demonstrated its clinical manifestations, specifically detailed audiological features in the Korean pediatric population.

## 2. Materials and Methods

### 2.1. Patients

A total of 38 patients diagnosed with cCMV infection were recruited in Seoul National University Hospital (SNUH) and Seoul National University Bundang Hospital (SNUBH). Children were ascertained either by clinically driven diagnosis (45%) or as a part of routine newborn CMV screening process in the neonatal intensive care unit (NICU) at SNUH (26%), or as a referral to the SNUBH ENT clinic for cochlear implant (CI) (29%). Congenital CMV infection was diagnosed via urine PCR identification of the virus DNA within the first two weeks of life in infants.

Patients who were diagnosed based on clinical suspicion between 2005 and 2016 were enrolled as having been clinically diagnosed with cCMV infection (group 1, n=17 from case 1-1 to 1-17). Patients with positive results from CMV screening in neonates born at or below 32 weeks/ or less than 1.5kg at birth (SNUH NICU protocol) were recruited from the NICU between 2014 and 2016 (group 2, n=10 from 2-1 to 2-10). Additionally, eleven patients with confirmed cCMV infection from another hospital, who had undergone CI in SNUBH, were also enrolled (group 3, n=11 from 3-1 to 3-11). These three groups were mutually exclusive. A retrospective study of 27 patients (groups 1 and 2) was performed to review the medical records, including the clinical and laboratory test results, hearing outcomes evaluated by multiple modalities, and any association with other developmental delays. As for group 3, given that their detailed medical documents regarding the diagnosis of cCMV infection at another hospital were unavailable and their audiologic characteristics were uniformly profound deafness, they were only analyzed for the postoperative CI outcomes and progression of hearing loss, which were collected from SNUBH medical archives.

This retrospective cohort study was approved by the institutional review boards of SNUBH (no. B-1612/376-106) and SNUH (no. J-1704-109-847).

### 2.2. Audiologic Assessment

Audiologic evaluations were made by Auditory Brainstem Response (ABR), Automated ABR (AABR), Pure Tone Audiometry (PTA), and Otoacoustic Emissions (OAE), including both Distortion Product OAE (DPOAE) and Transiently Evoked OAE (TEOAE). AABR was used for newborn hearing screening. Hearing level by threshold was classified as normal (0-25 dB), mild hearing loss (26-45 dB), moderate hearing loss (46-70 dB), severe hearing loss (71-90 dB), and profound hearing loss (≥91dB) [10–12]. Hearing threshold was based on all ears that could be evaluated, and the classification system was in accordance with the threshold of “worse ear.” Hearing deterioration/improvement was defined as an increase/decrease of ≥10 dB in the auditory threshold on consecutive hearing assessments and a change in hearing category. A difference between left and right ear auditory threshold* >*20 dB was indicated as asymmetric. The pre- and postoperative CI outcomes were evaluated with the Category of Auditory Performance (CAP), which is a global outcome measure of auditory abilities [13].

### 2.3. Statistical Analysis

The data are expressed as the means ± SD. Mann–Whitney test was used to compare the means of postoperative speech perception outcomes at each time point with preoperative speech perception outcome, and Fisher's exact test was performed to examine the association of risk factors with SNHL. Statistical analyses were performed and graphs were plotted with GraphPad prism 5.0 software (GraphPad Software, La Jolla, CA, USA).* P* < 0.05 was deemed to indicate statistical significance.

## 3. Results

### 3.1. Demographics of 38 Children with cCMV Infection (Groups 1, 2, and 3)

Among 27 patients diagnosed with cCMV infection (groups 1 and 2), fifteen patients were male (55.6%) and twelve patients were female (44.4%), and mean follow-up time was 39.8 months, ranging between 1 and 145 months. Demographics, including prematurity, low birth weight, hyperbilirubinemia requiring exchange transfusion, and NICU care for greater than 5 days are summarized (Table 1). Group 3 was composed of 8 males and 3 females. All patients from this group had abnormal newborn hearing screening (NBHS) results, except one patient who initially had passed NBHS and developed hearing loss at age 2 years.

To investigate the incidence of cCMV infection, we used the data from group 2, which was prospectively recruited according to the NICU protocol. Group 2 was selected among 276 screened neonates who were born at or below 32 weeks/ or less than 1.5kg at birth, resulting in an incidence rate of 3.6% for cCMV infected neonates meeting such criteria.

### 3.2. Audiologic Evaluations (Groups 1 and 2)

**Figure 1:**
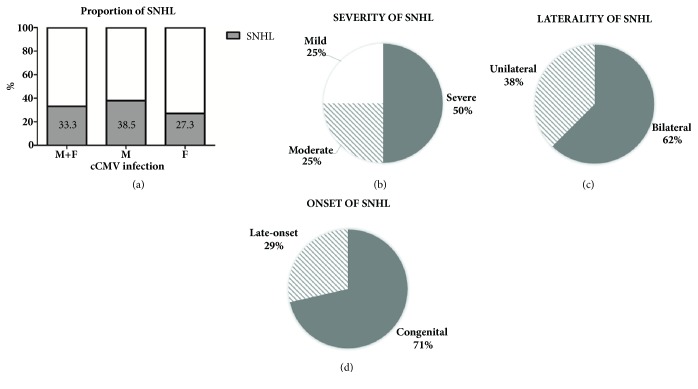
**Audiologic phenotypes of 8 patients with CMV-related SNHL**. (a) Proportion of SNHL in cCMV patients according to gender. (b) Classification of SNHL according to severity. (c), (d) Pie charts showing laterality and onset of SNHL.

Among 27 patients, hearing assessment by AABR, ABR, OAE, or PTA was performed in 24 patients, where the NBHS was performed in 21 patients. Among 3 patients who were not evaluated in NBHS, one showed hearing loss at first hearing evaluation at the age of 7 months (1-2). Two patients who passed the NBHS developed hearing loss at 2- and 7-month-old (1-4 and 1-5); the rate of late-onset hearing among CMV-related SNHL was 29% (2/7) in our cohort. In total, eight out of 24 patients presented hearing loss; the proportion of hearing loss in Korean cCMV infection patients was calculated to be 33.3% (8/24) (Table 2). However, an underestimation of CMV-related hearing loss in our cohorts may be possible because none of the 16 presumed normal hearing infants were audiologically followed up for more than 5 years after the birth. Audiologic phenotypes of eight patients with SNHL were further analyzed, not guaranteeing any pathognomonic audiometric features for CMV-related SNHL (Figure 1).

### 3.3. Consideration of Other Etiologies of SNHL and Antiviral Treatment (Groups 1 and 2)

No patient had severe hyperbilirubinemia that required exchange transfusion, and seven hearing-impaired patients whose OAE results were available demonstrating abnormal OAE findings, consistent with increased ABR thresholds, which excluded the possibility of auditory neuropathy spectrum disorder due to severe hyperbilirubinemia (Tables 1 and 2).

Sixteen patients out of 27 patients received antiviral therapy by intravenous Ganciclovir or oral Valganciclovir, or both. Among them, one patient suffered from bone marrow suppression, although his viral status became negative after a 3-week administration of IV Ganciclovir. Another patient had ganciclovir permanently discontinued due to severe neutropenia. Among eight patients with hearing loss, seven patients received antiviral therapy; three patients showed improved hearing while 2 patients demonstrated no change, and, for the remaining two patients, follow-up hearing test result was not available (Table 2). Notably, no antiviral recipient experienced deterioration of hearing.

### 3.4. Progression of Hearing Loss (Groups 1 and 3)

Regarding the progression of hearing loss, sixteen patients, comprising 5 patients from group 1 and 11 patients from group 3, with available follow-up audiologic assessments, were analyzed (Table 3). Three patients (1-1, 1-4, 1-5) showed gradual improvement, and six patients (3-1, 3-2, 3-5, 3-6, 3-7, 3-11) with profound deafness at the initial ABR remained unchanged, requiring CIs. Interestingly, four (3-4, 3-8, 3-9, 3-10) out of 11 CI implantees (group 3) demonstrated asymmetric hearing loss with no response on one side (at 100 dB stimulus). Initially they showed aidable hearing at one side with 50, 50, 55, and 75 dB of hearing threshold, but they rapidly progressed to profound hearing loss without exception before the age of 5 years, eventually ending up with bilateral CIs (Table 3). On the contrary, hearing status of two patients (1-6, 1-7) with asymmetric hearing loss with one side within normal range (single-sided deafness), remained unchanged, not necessarily requiring CI.

### 3.5. Outcomes of Cochlear Implantation in cCMV Patients (Group 3)

**Figure 2:**
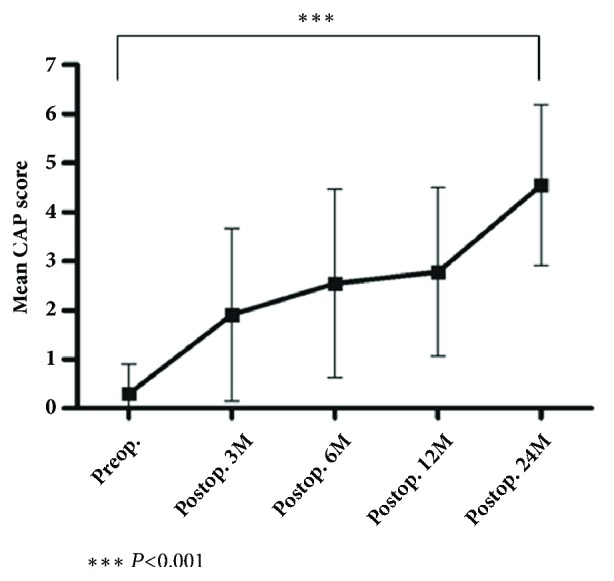
**Comparison of mean CAP scores at each time point before and after CI**. The mean CAP score gradually improved after CI during the observation period, with statistical significance (*P*=0.0013, 0.0006, respectively, at mean CAP score of 6mo and 12mo compared to preoperative score).

The outcome of CI for the 11 patients from group 3 was analyzed. The mean age of first implantation was 2.5±0.5 years, and six patients had bilateral implantations with 3 simultaneous CIs. Most patients, except one, with concurrent developmental delay showed significant improvement in speech perception, with an average CAP score that ranged from 0.3±0.6 preoperatively to 4.5±1.6 at 24 months after surgery (*P *< 0.0001) (Figure 2).

## 4. Discussion

Our Korean cohort showed that 33.3% of patients with cCMV infection had SNHL, 38% of asymmetric hearing loss, 29% of late-onset hearing loss, and a diverse spectrum of SNHL severity, ranging from mild to profound among CMV-related SNHL patients. Interestingly, there was no hearing-impaired patient in group 2. However, this should be interpreted very cautiously given the relatively high incidence of delayed-onset hearing loss among cCMV infection patients, with the onset mostly within 5 years of birth [14]. The incidence of cCMV infection in our cohort was 3.6%, which was higher than figures previously reported in other studies [9, 15]. However, given the characteristics of group 2 with mostly premature births in the NICU, higher incidence is likely. Among the sixteen CMV-related hearing loss patients, whose audiological follow-up was available, four patients (3-4, 3-8, 3-9, 3-10) with initial asymmetric hearing loss, progressed to profound deafness before the age of 5 years, while two patients (1-6, 1-7) with single-sided deafness, maintained healthy hearing in the follow-up hearing evaluation. In case of asymmetric hearing loss, it seemed that when the better side hearing threshold was over 50 dB while the other side showed no response at ABR test, this condition would eventually aggravate to the profound hearing loss. This speculation might provide clinical insight into counseling these patients to receive CI on the worse side first, then wait to see the hearing course of the other side for better auditory rehabilitation. Fortunately, patients with CMV-related hearing loss showed favorable speech perception after CI, in agreement with previous studies in other ethnicities [16, 17], making Korean patients with CMV-related SNHL also good candidates for CI.

Several risk factors for CMV-related deafness were proposed by the Joint Committee on Infant Hearing. Among the risk factors, severe neonatal jaundice was shown to cause neurotoxicity, impairing the auditory system, which is known to be highly sensitive to overt bilirubin-induced toxicity [18, 19]. Infant hearing loss associated with severe jaundice was known to cause auditory neuropathy spectrum disorder (ANSD), characterized by abnormal or absent ABR, but normal OAE [20, 21]. In our study, all hearing-impaired patients with available OAE data had abnormal OAE findings, consistent with abnormal ABR results, which could exclude the possibility of jaundice-related ANSD; at least in our cohort, the CMV etiology of SNHL was supported. Other risk factors, including long NICU stay, prematurity, and low birth weight, showed no predisposition to hearing loss in our study. On the contrary, prematurity and very low birth weight showed an association with normal hearing in CMV patients (*P*=0.006 and* P*=0.008, respectively) as opposed to the previous study [3]. However, this discrepancy may be due to the high number of NICU patients in our study population and a robust conclusion also could not be drawn due to a limitation stemming from a small number of each group.

Our study has several limitations. We included three groups with distinct demographic features. Hence, our study may suffer from selection bias, hindering the generalizability. For example, patients in group 2 were recruited from the NICU, which included more preterm patients. Therefore, when analyzing our data, we used each group selectively to reduce selection bias. Moreover, due to the nature of retrospective study, incidence of cCMV infection and CMV-related hearing loss could not be deduced accurately, limiting the usefulness of the epidemiologic data.

## 5. Conclusion

CMV turned out to be a significant cause of neonatal and infant SNHL, first to date reported in the Korean population. Our analysis showed diverse audiologic features but with favorable CI outcomes. Moreover, in our study, three patients developed late-onset SNHL after passing NBHS, and four patients with asymmetric audiologic configuration progressed to bilateral profound deafness as late as the age of 5 years. This indicates that periodic audiologic evaluations should be mandatory, at least up to early childhood, for those with positive CMV results, regardless of the NBHS results. Additionally, for those who have asymmetric hearing loss with one side of profound hearing loss, we could advise them to consider CI on the worse side first without delay, eventually benefitting the patient from the CI for better auditory rehabilitation. We hope that the findings highlighted in this study could help physicians to acquire better understanding of audiologic phenotypes of CMV-related hearing loss according to a particular clinical setting.

## Figures and Tables

**Table 1:** Demographics of patients with cCMV infection (Groups 1 and 2).

	Present (*N* of patients with SNHL)	Absent (*N* of patients with SNHL)
Prematurity	18 (2)	9 (6)
Very Low birth weight (<1500g)	15 (1)	12 (7)
Hyperbilirubinemia requiring exchange transfusion	0	21 (8)
NICU stay greater than 5 days	22 (6)	5 (2)
Hearing loss at birth	5	16

Prematurity: neonates born at less than 37 weeks' gestation

**Table 2:** Characteristics of hearing loss observed in patients with cCMV-related SNHL (Group 1).

Case	NBHS	Sex	Laterality	Severity	Onset	Initial ABR (dB)	Follow-up ABR or Audio (dB)	OAE	Progression	Antiviral therapy	Preterm birth
1-1	Refer	M	Bilateral	Severe	Cong.	B)NR	B)40	Abnl.	Improved	Done	Yes
1-2	N/A	M	Bilateral	Mild	Unclear	B)30		Abnl.	N/A	Done	Yes
1-3	Refer	M	Bilateral	Severe	Cong.	R)NR, L)70		Abnl.	N/A	Not done	No
1-4	Pass	M	Unilateral	Mild	2 mo.	R)25, L)35	R)15, L)12	Abnl.	Improved	Done	No
1-5	Pass	F	Bilateral	Moderate	7 mo.	R)55, L)30	B)35	Abnl.	Improved	Done	No
1-6	Refer	F	Unilateral	Severe	Cong.	R)25, L)NR	R)25, L)NR	Abnl.	No change	Done	No
1-7	Refer	M	Unilateral	Severe	Cong.	R)NR, L)20	R)NR, L)20	Abnl.	No change	Done	No
1-8	Refer	F	Bilateral	N/A	Cong.	N/A		N/A	N/A	Done	No

Abnl: abnormal; Cong.: congenital; NR: no response; mo.: month-old; N/A: not available

**Table 3:** Progression of hearing loss observed in patients with cCMV-related SNHL (Groups 1 and 3).

Case	NBHS	Sex	Laterality	Severity	Onset	Initial ABR (dB)	Follow-up ABR or Audio (dB)	Progression
1-1	Refer	M	Bilateral	Severe	Cong.	B)NR	B)40	Improved
1-4	Pass	M	Unilateral	Mild	2 mo.	R)25, L)35	R)15, L)10	Improved
1-5	Pass	F	Bilateral	Moderate	7 mo.	R)55, L)30	B)35	Improved
1-6	Refer	F	Unilateral	Severe	Cong.	R)25, L)NR	R)25, L)NR	No change
1-7	Refer	M	Unilateral	Severe	Cong.	R)NR, L)20	R)NR, L)20	No change
3-1	Refer	F	Bilateral	Profound	Cong.	B)NR	R)100, L)100	No change
3-2	Pass	M	Bilateral	Profound	26 mo.	B)NR	R)100, L)100	No change
3-3	Refer	M	Bilateral	Profound	Cong.	R)90, L)NR	R)115, L)110	Aggravated
3-4	Refer	M	Bilateral	Profound	Cong.	R)50, L)NR	R)90, L)90	Aggravated (Asymmetric to symmetric profound)
3-5	Refer	M	Bilateral	Profound	Cong.	B)NR	R)100, L)110	No change
3-6	Refer	M	Bilateral	Profound	Cong.	B)NR	B)115	No change
3-7	N/A	F	Bilateral	Profound	Unclear	B)NR	R)95, L)105	No change
3-8	Refer	M	Bilateral	Profound	Cong.	R)70, L)NR	R)90, L)100	Aggravated (Asymmetric to symmetric profound)
3-9	Refer	M	Bilateral	Profound	Cong.	R)55, L)105	R)100, L)110	Aggravated (Asymmetric to symmetric profound)
3-10	Refer	F	Bilateral	Profound	Cong.	R)NR, L)50	B)100	Aggravated (Asymmetric to symmetric profound)
3-11	Refer	M	Bilateral	Profound	Cong.	B)NR	B)100	No change

Abnl: abnormal; Cong.: congenital; NR: no response; mo.: month-old; N/A: not available

## Data Availability

The data used to support the findings of this study are included within the article.
